# 伊布替尼治疗淋巴浆细胞淋巴瘤/华氏巨球蛋白血症的疗效及安全性

**DOI:** 10.3760/cma.j.cn121090-20240301-00077

**Published:** 2024-08

**Authors:** 燕姗 黄, 文婕 熊, 菁菁 原, 颖 于, 雨曦 李, 禹廷 阎, 婷玉 王, 瑞 吕, 薇 刘, 刚 安, 耀中 赵, 德慧 邹, 录贵 邱, 树华 易

**Affiliations:** 1 中国医学科学院血液病医院（中国医学科学院血液学研究所），实验血液学国家重点实验室，国家血液系统疾病临床医学研究中心，细胞生态海河实验室，天津 300020 State Key Laboratory of Experimental Hematology, National Clinical Research Center for Blood Diseases, Haihe Laboratory of Cell Ecosystem, Institute of Hematology & Blood Diseases Hospital, Chinese Academy of Medical Sciences & Peking Union Medical College, Tianjin 300020, China; 2 天津医学健康研究院，天津 301600 Tianjin Institutes of Health Science, Tianjin 301600, China

**Keywords:** Waldenstrom巨球蛋白血症, 淋巴瘤, 伊布替尼, 治疗结果, Waldenstrom macroglobulinemia, Lymphoma, Ibrutinib, Treatment outcome

## Abstract

**目的:**

探索伊布替尼治疗初治及复发难治（R/R）淋巴浆细胞淋巴瘤（LPL）/华氏巨球蛋白血症（WM）的疗效及安全性。

**方法:**

收集2016年3月至2023年6月在中国医学科学院血液病医院接受伊布替尼治疗的98例初治及R/R LPL/WM患者的临床资料，回顾性分析其疗效及安全性。

**结果:**

共纳入98例LPL/WM患者，初治患者45例，R/R患者53例，男74例（75.5％），中位年龄64（42～87）岁。88例患者可进行疗效评估，中位治疗时间20.8（2.1～55.0）个月，主要缓解率（MRR）为78.4％，总缓解率（ORR）为85.2％。初治患者的MRR和ORR分别为78.4％和86.5％，R/R患者的MRR和ORR分别为78.4％和84.3％，初治与R/R患者MRR和ORR的差异均无统计学意义（*P*值均>0.05）。中位随访29.1（2.9～50.3）个月，初治和R/R患者的中位总生存时间均未达到，中位无进展生存时间分别为23.5（95％*CI* 10.5～36.5）个月和45.0（95％*CI* 34.0～56.0）个月，差异均无统计学意义（*P*值均>0.05）。死亡患者25例，未出现因应用伊布替尼死亡事件，伊布替尼的主要不良反应为血小板减少（5.1％）、肺炎（8.1％）及高尿酸血症（21.4％），心房颤动的发生率为2.0％。

**结论:**

伊布替尼对于初治及R/R LPL/WM患者有良好的疗效及安全性。

## Abstract (en)

**Objective:**

To explore the efficacy and safety of ibrutinib for the treatment of newly treated and relapsed refractory（R/R）lymphoplasmacytic lymphoma（LPL）/Waldenström macroglobulinemia（WM）.

**Methods:**

Retrospectively collected clinical data of 98 cases of newly treated and R/R LPL/WM patients who received ibrutinib treatment at the Hematology & Blood Diseases Hospital of the Chinese Academy of Medical Sciences from March 2016 to June 2023, and analyzed their efficacy and safety.

**Results:**

A total of 98 LPL/WM patients were included, which consisted of 45 newly treated patients and 53 R/R patients. Of these, 74 were males（75.5％）and the cohort had a median age of 64（42–87）years. Eighty-eight patients were eligible for efficacy evaluation with a median treatment time of 20.8（2.1–55.0）months, a major remission rate（MRR）of 78.4％, and an overall response rate（ORR）of 85.2％. The MRR and ORR of the newly treated patients were 78.4％ and 86.5％, respectively, whereas the MRR and ORR of the R/R patients were 78.4％ and 84.3％, respectively. There were no statistically significant differences in MRR and ORR between the initial treatment and R/R patients（all *P* values>0.05）. The median follow-up period was 29.1（2.9–50.3）months and the median overall survival time for newly treated and R/R patients was not reached. The median progression-free survival time was 23.5（95％ *CI* 10.5–36.5）months and 45.0（95％ *CI* 34.0–56.0）months, respectively, with no statistically significant differences（all *P* values >0.05）. There were 25 deceased patients and no deaths were related to ibrutinib treatment. The main adverse reactions of ibrutinib were thrombocytopenia（5.1％）, pneumonia（8.1％）, and hyperuricemia（21.4％）. The incidence of atrial fibrillation was 2.0％.

**Conclusion:**

Ibrutinib exhibits good efficacy and safety for newly treated and R/R LPL/WM patients.

淋巴浆细胞淋巴瘤（Lymphoplasmacytic lymphoma，LPL）是一种罕见的惰性B细胞淋巴瘤，其特征是克隆性淋巴浆细胞浸润骨髓和分泌单克隆免疫球蛋白。90％～95％的LPL分泌单克隆性IgM，为华氏巨球蛋白血症（Waldenström macroglobulinemia，WM）[1]，髓样分化因子88（MYD88）是白细胞介素-1和Toll样受体信号传导复合物的组成成分，MYD88^L265P^突变能促进淋巴浆细胞的恶性增殖[2]。约90％以上和30％～35％的WM患者伴MYD88和趋化因子受体4（CXCR4）的体细胞突变[3]。伊布替尼作为第一代布鲁顿酪氨酸激酶（BTK）抑制剂（BTKi），已成为WM患者的标准治疗。然而，目前中国尚缺乏伊布替尼治疗初治及复发难治（R/R）WM患者的研究，且不同疾病状态患者接受伊布替尼治疗后疗效差异尚不明确。本研究对98例接受伊布替尼治疗的初诊及R/R症状性LPL/WM患者进行回顾性分析，旨在探索伊布替尼的疗效及安全性。

## 病例与方法

1. 病例：纳入2016年3月至2023年6月中国医学科学院血液病医院符合治疗指征并接受伊布替尼治疗的98例LPL/WM患者，其中单药治疗84例（85.7％），联合治疗14例（14.3％）。所有患者均符合LPL/WM的诊断标准[4]–[5]。参照WM国际预后评分系统（ISSWM）进行危险分层[6]。

2. MYD88^L265P^和CXCR4突变检测：采用一代测序（Sanger测序）、等位基因特异性聚合酶链反应（AS-PCR）、数字液滴PCR（dd-PCR）和二代测序（NGS）对MYD88^L265P^突变进行检测，采用Sanger测序、AS-PCR和NGS对CXCR4进行检测。只要其中一项检测阳性，则定义为基因突变阳性。标本来源包括骨髓（91.8％）、外周血（6.1％）、其他（2.0％）。

3. 疗效和安全性评价：疗效评价主要参考国际WM工作组推荐的疗效评价标准[7]。主要缓解率（MRR）定义为获得部分缓解（PR）及以上疗效患者所占比例，总缓解率（ORR）定义为获得微小缓解（MR）及以上疗效患者所占比例。最佳疗效为治疗及随访期间任何一次评估中的最佳疗效。总生存（OS）时间指患者开始接受伊布替尼治疗至因任何原因死亡或末次随访的时间，失访患者以末次随访状态为截止数据。无进展生存（PFS）时间指患者开始接受伊布替尼治疗至疾病进展、复发、死亡的时间，PFS2定义为患者自接受二线治疗至疾病进展、死亡的时间。治疗时间指患者开始接受伊布替尼治疗至任何原因导致停药的时间。根据美国国家癌症研究所制定的常见不良事件评价（NCI CTCAE）5.0版对不良事件（AE）进行分级。

4. 随访：采用电话联系的方式随访。随访时间截至2023年10月18日。随访内容包括患者的血常规、免疫球蛋白、免疫固定电泳、淋巴结超声及腹部超声结果等。

5. 统计学处理：采用IBM SPSS Statistics 26.0及GraphPad Prism 9.4.1软件进行统计分析。计量资料用中位数（范围）表示，计数资料用例数（百分比）表示。组间比较采用*χ*^2^检验，采用Kaplan-Meier法分析患者的生存情况，*P*<0.05为差异有统计学意义。

## 结果

1. 基线特征：本研究纳入98例患者，中位年龄64（42～87）岁，初治患者45例，R/R患者53例。其中，二线应用伊布替尼治疗的患者36例，三线及以上应用伊布替尼患者17例。男74例（75.5％），中位免疫球蛋白水平为34.2（1.1～144.0）g/L，IgM型89例（90.8％），IgG型6例（6.1％）。初治患者ISSWM评分低、中、高危组分别为3例（7.7％）、12例（30.7％）和24例（61.5％）；R/R患者ISSWM评分低、中、高危组分别为15例（28.3％）、20例（37.7％）和18例（34.0％）。98例患者中MYD88^L265P^突变患者90例（91.8％），CXCR4突变患者37例（37.7％）。初治和R/R组年龄>65岁患者比例、ISSWM评分及骨髓受累比例的差异有统计学意义（表1）。

**表1:** 接受伊布替尼治疗的复发难治及初治LPL/WM患者的临床特征比较［例（％）］

基线特征	复发难治（53例）	初治（45例）	*χ*^2^值	*P*值
年龄>65岁	20（37.7）	26（57.8）	3.93	0.048
男性	42（79.3）	32（71.1）	0.87	0.351
ISSWM评分			8.93	0.011
低危	15（28.3）	3（7.7）		
中危	20（37.7）	12（30.8）		
高危	18（34.0）	24（61.5）		
IgM			0.01	0.928
≥40 g/L	20（37.7）	17（38.6）		
<40 g/L	33（62.3）	27（61.4）		
β_2_-微球蛋白>3 mg/L	32（72.7）	33（84.2）	1.83	0.186
MYD88/CXCR4基因类型			0.89	0.829
MYD88^L265P^/CXCR4^WT^	26（52.0）	23（54.8）		
MYD88^L265P^/CXCR4^MUT^	20（40.0）	14（33.3）		
MYD88^WT^/CXCR4^WT^	3（6.0）	3（7.1）		
MYD88^WT^/CXCR4^MUT^	1（2.0）	2（4.8）		
染色体核型			0.16	0.687
复杂核型^a^	18（42.9）	15（38.5）		
正常核型	24（57.1）	24（61.5）		
骨髓受累	46（87.8）	30（66.7）	5.66	0.017
淋巴结肿大	20（54.1）	15（36.6）	2.06	0.121
脾肿大	24（52.2）	16（43.2）	0.39	0.418
HGB≤110 g/L	38（71.7）	37（2.2）	1.50	0.221
PLT≤100×10^9^/L	16（30.2）	13（28.9）	0.02	0.888
中性粒细胞绝对值≤1.5×10^9^/L	11（22.0）	5（13.9）	0.91	0.198

**注** LPL：淋巴浆细胞淋巴瘤；WM：华氏巨球蛋白血症；ISSWM：WM国际预后评分系统；^a^复杂核型：≥3种染色体异常

2. 疗效：98例患者中，初治及R/R患者中位治疗时间分别为28.2（2.1～55.0）个月和28.3（5.7～46.9）个月，中位起效时间分别为1.8（0.7～13.0）个月和1.6（0.5～7.1）个月。88例（89.8％）可进行疗效评价的患者中，8例（9.1％）患者疗效达到了非常好的部分缓解（VGPR）及以上。总体MRR为78.4％，ORR为85.2％。初治患者的MRR和ORR分别为78.4％和86.5％；R/R患者的MRR和ORR分别为78.4％和84.3％，两组MRR和ORR的差异均无统计学意义（*P*值均>0.05）。接受2线和≥3线治疗患者的MRR分别为85.3％和64.7％，ORR分别为88.2％和76.5％，MRR和ORR差异均无统计学意义（*P*值均>0.05）。所有接受治疗的患者HGB及免疫球蛋白水平均明显改善。治疗前，42例（42.8％）患者HGB<90 g/L，治疗后30例患者HGB恢复正常，治疗前后中位HGB水平分别为92（61～132）g/L和133（102～157）g/L；治疗前，免疫球蛋白≥40 g/L患者37例（37.8％），伊布替尼治疗后，免疫球蛋白中位水平下降了73.3％，且在随访期间血清IgM水平保持稳定。

进一步探讨MYD88及CXCR4基因突变对患者疗效的影响。根据基因突变结果将患者分为MYD88^L265P^CXCR4^WT^组和MYD88^L265P^CXCR4^MUT^组。MYD88^L265P^CXCR4^WT^组44例患者可进行疗效评价，其中7例（15.9％）患者达VGPR及以上疗效，MRR为77.3％，ORR为86.4％。MYD88^L265P^CXCR4^MUT^组31例患者可进行疗效评价，其中1例（3.2％）患者达VGPR以上疗效，MRR为80.7％，ORR为87.1％。MYD88^L265P^CXCR4^MUT^组与MYD88^L265P^CXCR4^WT^组MRR、ORR及VGPR率的差异均无统计学意义（*P*值分别为0.927、0.726和0.080）。将相同基因型的患者根据初治及R/R进行分类，初治患者MYD88^L265P^CXCR4^WT^组和MYD88^L265P^CXCR4^MUT^组的MRR分别为84.2％和72.7％（*P*>0.05），ORR分别为94.7％和81.8％（*P*>0.05）。R/R患者MYD88^L265P^CXCR4^WT^组和MYD88^L265P^CXCR4^MUT^组的MRR分别为72.0％和85.0％（*P*>0.05），ORR分别为80.0％和95.0％（*P*>0.05）。

对疗效达到PR以上的患者进行单因素分析。结果显示，CD23不表达和复杂核型对深度缓解有明显影响（*P*值分别为0.042、0.040）。LDH升高、治疗时间对初治患者的疗效有显著影响（*P*值分别为0.011、0.021）。LDH升高、复杂核型和治疗时间是R/R患者深度缓解的影响因素（*P*值分别为0.003、0.032和0.028）。

3. 生存：中位随访29.1（2.9～50.3）个月，初治和R/R患者的中位OS时间均未达到（*P*＝0.768）。初治和R/R患者的中位PFS时间分别为23.5（95％*CI* 10.5～36.5）个月和45.0（95％*CI* 34.0～56.0）个月，（*P*<0.001）（图1）。初治患者和R/R患者的中位起效时间分别为1.8（0.7～13.0）个月和1.6（0.5～7.1）个月。进一步分析不同治疗线数患者的生存情况，1线、2线和≥3线患者的中位OS时间均未达到（*P*＝0.273），中位PFS时间分别为34.0个月、未达到和19.4个月（*P*＝0.043）（图2）。

**图1:**
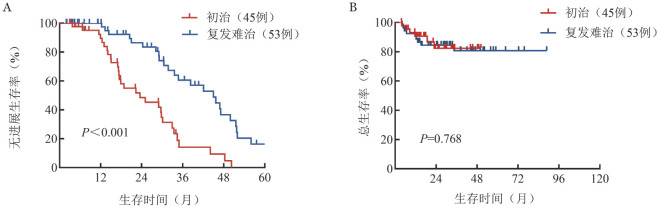
初治和复发难治淋巴浆细胞淋巴瘤/华氏巨球蛋白血症患者的无进展生存（A）和总生存（B）曲线

**图2:**
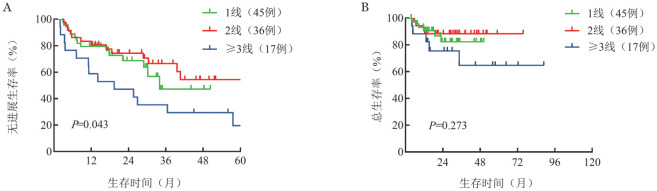
不同治疗线数淋巴浆细胞淋巴瘤/华氏巨球蛋白血症患者的无进展生存（A）和总生存（B）曲线

根据不同突变类型进行分析，发现CXCR4^MUT^患者早期死亡率较高，其中OS时间<12个月的患者中，CXCR4^MUT^患者9例（64.3％）。对于相同基因型的初治及R/R患者，OS和PFS的差异均无统计学意义（*P*值均>0.05）。MYD88^L265P^CXCR4^WT^基因类型的初治和R/R患者的中位OS时间均未达到，中位PFS时间分别为未达到和57.6个月；而MYD88^L265P^CXCR4^MUT^基因类型的初治和R/R患者的中位OS时间均未达到，中位PFS时间分别为30.2个月和未达到。进一步对不同基因型的初治及R/R患者进行生存分析，初治患者中MYD88^L265P^CXCR4^MUT^、MYD88^L265P^CXCR4^WT^及MYD88^WT^患者的中位OS时间均未达到（*P*值均>0.05），而PFS时间分别为未达到、30.2个月和23.4个月（*P*＝0.041）；对于R/R患者，MYD88^L265P^CXCR4^MUT^、MYD88^L265P^CXCR4^WT^及MYD88^WT^患者的中位OS时间同样均未达到（*P*值均>0.05），而PFS时间分别为57.6个月、未达到和18.6个月（*P*>0.05）。

单因素分析显示，疗效达PR及以上、治疗时间、CD23不表达及复杂核型对PFS有明显影响（*P*值分别为0.001、<0.001、0.004、0.043），但CD23不表达及复杂核型对OS无明显影响。值得注意的是，治疗时间对患者的PFS及OS均有明显影响（*P*值均<0.001），其中治疗时间>24个月的患者PFS及OS时间明显延长（图3）。

**图3:**
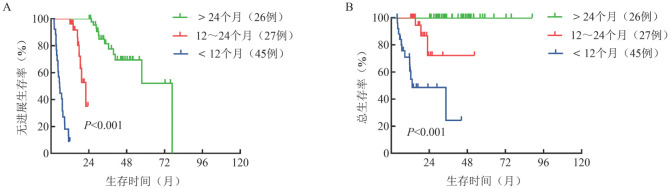
不同治疗时间淋巴浆细胞淋巴瘤/华氏巨球蛋白血症患者的无进展生存（A）和总生存（B）曲线

4. 安全性：接受伊布替尼治疗的98例患者中，42例（42.9％）患者出现进展/死亡。其中初治患者15例，R/R患者27例。在初治患者中，9例在治疗未满12个月时出现早期进展/死亡，其中4例因本病死亡，最短治疗时间2.9个月；在R/R患者中，13例在治疗未满12个月时出现早期进展/死亡，其中7例因本病死亡。所有患者均未出现治疗相关死亡。伊布替尼治疗过程中最常见的不良反应为血小板减少（5.1％）、肺炎（8.2％）及高尿酸血症（21.4％）。在98例患者中，共9例（9.2％）患者出现血液学不良反应，5例为Ⅲ/Ⅳ级，其中2例为初治患者，均为血小板减少；3例为R/R患者，分别为中性粒细胞减少、贫血、血小板减少。14例（14.3％）出现Ⅲ/Ⅳ级非血液学不良反应，包括肺炎4例、周围神经损害2例、高尿酸血症7例及高黏滞血症1例（表2）。

**表2:** 初治及复发难治（R/R）淋巴浆细胞淋巴瘤/华氏巨球蛋白血症患者应用伊布替尼的不良反应［例（％）］

不良反应	总体		≥3级	
	R/R	初治	R/R	初治
血液学不良反应				
中性粒细胞减少	2（2.0）	0（0）	1（1.0）	0（0）
贫血	1（1.0）	1（1.0）	1（1.0）	0（0）
血小板减少	3（3.1）	2（2.0）	1（1.0）	2（2.0）
非血液学不良反应				
周围神经损害	3（3.1）	0（0）	2（2.0）	0（0）
高黏滞血症	2（2.0）	1（1.0）	1（1.0）	0（0）
心律失常	1（1.0）	1（1.0）	0（0）	0（0）
肺炎	5（5.1）	3（3.1）	3（3.1）	1（1.0）
高尿酸血症	6（6.1）	15（15.3）	3（3.1）	4（4.1）

## 讨论

伊布替尼对于症状性初治及R/R LPL/WM患者有显著疗效，已被国内外多项研究证实为一线治疗方案。本研究结果显示，初治及R/R LPL/WM患者OS的差异无统计学意义，但R/R患者的PFS明显优于初治患者，与Treon等[7]–[8]的研究结果存在差异。研究发现，初治WM患者中位接受伊布替尼单药治疗13.4个月的MRR和ORR分别为83.0％和100.0％，18个月OS率及PFS率分别为100.0％和92.0％[7]；R/R WM患者接受伊布替尼单药治疗的MRR和ORR分别为79.4％和90.5％，5年OS率及PFS率分别为87.0％和54.0％[8]。在上述两项研究中[7]–[8]，初治患者的治疗有效率优于本研究，而R/R患者的治疗有效率未见明显差异。原因可能与患者的异质性、治疗方案的多样性及随访时间等因素有关。值得注意的是，对于R/R患者而言，早期应用伊布替尼似乎能获得更高的深度缓解率，OS虽然未见明显差异，但PFS具有显著优势。

研究显示，BTKi治疗的时间与生存显著相关[9]，与本研究结果一致，单因素分析显示伊布替尼治疗时间是生存的影响因素，提示提高患者依从性、确保患者长期接受口服治疗的重要性。

MYD88^L265P^和CXCR4的突变情况均对疗效和生存有显著影响。本研究中MYD88^L265P^的突变率为91.8％，与国外研究报道结果一致[3],[10]，与国内报道相比突变率偏高[11]–[13]。研究发现，骨髓样本的检出率高于外周血及淋巴结等样本[13]，此外，本研究应用NGS及AS-PCR等方法进行检测，提高了检出率。MYD88及CXCR4基因突变情况是BTKi疗效及预后的重要参考因素，MYD88^WT^患者在接受治疗后均未获得深度缓解，可能与MYD88^WT^患者具有BTK NF-κB的激活突变有关[14]。此外，CXCR4的突变状态对患者的缓解深度有影响，CXCR4^WT^患者深度缓解率更高，达到VGPR及以上疗效的患者更多，相关研究也报道了类似结果[3],[5],[13],[15]。在初治患者中CXCR4^WT^患者的MRR和ORR较CXCR4^MUT^患者高，R/R患者中CXCR4^MUT^患者的MRR和ORR高于CXCR4^WT^患者，但差异无统计学意义，本研究初治及R/R患者中CXCR4^MUT^患者的比例相当，可能与样本数较少相关，期待未来更大样本量数据的验证。

伊布替尼在本研究中展现出良好的疗效及安全性。尽管有研究表明接受伊布替尼治疗的WM患者最常见的不良反应为心房颤动、室性心律失常和高血压等[5],[16]，但本研究中最常见的不良反应为血小板减少、高尿酸血症及肺炎，心律失常的发生率为2.0％，且均未达Ⅲ级，与本中心既往研究的结果一致[9]。本研究中，11例患者出现早期进展后死亡，其中CXCR4^MUT^患者9例，早期进展死亡可能与CXCR4突变相关，该突变会导致患者的骨髓受累程度加重、IgM水平升高、高黏滞血症，诊断时疾病更具侵袭性。此外，会降低伊布替尼治疗的敏感性[17]，但仍需进一步研究证实。

尽管本研究是中国最大系列伊布替尼治疗WM的真实世界研究，具有一定的参考价值，但仍有不足之处。由于联合治疗的例数较少，本研究未能进行伊布替尼单药及联合治疗的分层分析。本中心既往发表的研究表明BTKi单药及联合治疗B细胞淋巴增殖性疾病的疗效未见显著差异[9]。

综上所述，伊布替尼对于LPL/WM有良好的疗效，可以长期控制疾病进展。治疗的疗效受MYD88和CXCR4突变状态、治疗时间及治疗线数的影响。但总体而言，治疗的耐受性良好，无不可控制的不良反应，应用伊布替尼治疗LPL/WM是安全有效的。
